# Differential effects of computerized cognitive stimulation versus stimulating leisure activities on mood and global cognition in adults with subjective and mild cognitive impairment: A randomized controlled trial

**DOI:** 10.23938/ASSN.1136

**Published:** 2025-11-25

**Authors:** Isabel Gómez-Soria, Juan Nicolás Cuenca-Zaldivar, Bárbara Oliván-Blázquez, Alejandra Aguilar-Latorre, Rosa Mª Magallón-Botaya, Estela Calatayud

**Affiliations:** 1 University of Zaragoza Faculty of Health Sciences Department of Physiatry and Nursing Zaragoza Spain; 2 Institute for Health Research Aragón (IIS Aragón) Zaragoza Spain; 3 Primary Health Center “El Abajon” Las Rozas Madrid Spain; 4 Grupo de Investigación en Enfermería y Cuidados de Salud Instituto de Investigación Sanitaria Puerta de Hierro - Segovia de Arana (IDIPHISA) Madrid España; 5 Fundación para la Investigación e Innovación Biosanitaria en Atención Primaria (FIIBAP) Madrid España; 6 University of Zaragoza Faculty of Social and Labor Sciences Department of Psychology and Sociology Zaragoza Spain; 7 University of Zaragoza Faculty of Human Sciences and Education Department of Psychology and Sociology Huesca Spain; 8 University of Zaragoza Faculty of Medicine Department of Medicine, Psychiatry and Dermatology Zaragoza Spain

**Keywords:** Mild Cognitive Impairment, Subjective Cognitive Impairment, Computerized Cognitive Stimulation, Stimulating Leisure Activities, Primary Care, Deterioro Cognitivo Leve, Quejas Subjetivas de Memoria, Estimulación Cognitiva Computarizada, Actividades de Ocio Estimulantes, Atención Primaria

## Abstract

**Background:**

Mild Cognitive Impairment (MCI) involves both subjective and objective cognitive decline with relatively preserved daily functioning, whereas Subjective Cognitive Impairment (SCI) refers to perceived decline without measurable deficits. This study aimed to compare the effects of a personalized computerized cognitive stimulation (CS) program (IG1) and a stimulating leisure activities program (IG2) in adults with MCI and SCI.

**Methods:**

A single-blind randomized controlled trial was conducted in primary care with participants aged ≥50 years with SCI or MCI. Participants were randomized to IG1, IG2), or a control group (CG). IG1 completed personalized computerized CS for 30 minutes/day, 5 days/week, for 8 weeks. IG2 engaged in 2-5 stimulating leisure activities per week for the same period. The primary outcome was global cognition; secondary outcomes included memory, verbal fluency, daily functioning, and mood.

**Results:**

Fifty-nine participants were enrolled in the study, 44 with SCI and 15 with MCI. Compared to CG, IG1 showed greater reductions in anxiety post-intervention (2.07; 95%CI: 0.93-3.22 vs. 4.34; 95%CI: 3.22-5.46) and lower depressive symptoms at 6-month follow-up (3.41; 95%CI: 2.05-4.77 vs. 5.62; 95%CI: 4.26-6.97). IG2 participants demonstrated improved global cognition post-intervention (29.2; 95%CI: 27.625-30.776) and at 6 months (28.78; 95%CI: 27.16-30.42) relative to CG (30.626; 95%CI: 28.99-32.27).

**Conclusion:**

Personalized computerized CS reduces anxiety and depression symptoms, while stimulating leisure activities enhances global cognition in community-dwelling adults with SCI and MCI, suggesting complementary benefits of both interventions.

## RESUMEN

**Fundamento:**

El deterioro cognitivo leve (DCL) se caracteriza por deterioro cognitivo subjetivo y objetivo con funcionamiento diario relativamente preservado, mientras que el deterioro cognitivo subjetivo (DCS) implica percepción de deterioro cognitivo sin déficits medibles. Este estudio evalúa los efectos de un programa personalizado de estimulación cognitiva computarizada frente a actividades de ocio estimulantes en adultos con DCL y DCS.

**Métodos:**

Ensayo controlado aleatorizado simple ciego realizado en Atención Primaria con participantes de ≥50 años, con DCL o DCS, asignados aleatoriamente a dos grupos de intervención (GI1, GI2) y a un grupo control (GC). El GI1 realizó estimulación cognitiva computarizada personalizada 30 minutos/día, 5 días/semana, durante 8 semanas. El GI2 participó en 2-5 actividades de ocio estimulantes/semana durante el mismo periodo. El resultado principal fue la cognición global y los secundarios memoria, fluidez verbal, funcionamiento diario y estado de ánimo.

**Resultados:**

Se reclutaron 59 participantes, 15 con DCL. Frente al GC, el GI1 redujo más la ansiedad post-intervención (2,074; IC95%: 0,927-3,222 vs. 4,338; IC95%: 3.22-5.456) y los síntomas depresivos a los 6 meses (3,407; IC95%: 2,047-4,767 vs. 5,615; IC95%: 4,262-6,968), mientras que el GI2 mejoró la cognición global tanto post-intervención (29,2; IC95%: 27,625-30.776) como a los 6 meses (28,782; IC95%: 27,163-30,402) respecto del GC (30,626; IC95%: 28,987-32,265).

**Conclusión:**

La estimulación cognitiva computarizada personalizada redujo los síntomas ansiosos y depresivos, mientras que las actividades de ocio estimulantes mejoraron la cognición global en adultos con DCS y DCL no institucionalizados, sugiriendo beneficios complementarios de ambos enfoques.

## INTRODUCTION

The world’s population is ageing rapidly, placing substantial pressure on societies to adapt to shifting demographic needs^1^. As the population ages, concerns about cognitive impairment are becoming increasingly common in clinical practice, encompassing various degrees of cognitive and functional decline as evidenced by clinical and neuropsychological assessments^2^.

Mild cognitive impairment (MCI) is a clinical condition characterized by subjective and objective cognitive decline with a relative preservation of activities of daily living (ADL)^3^. Although individuals with MCI generally maintain functional independence^4^, subtle functional limitations may occur. Complex or instrumental ADL can become more effortful, time-consuming, or dependent on compensatory strategies^3^. Depression, anxiety, and apathy are highly prevalent among older adults with MCI and are important predictors of progression from MCI to dementia^5^. High-risk depressive symptoms are associated with an increased risk of subsequent MCI (SHR = 1.20; 95%CI: 1.08-1.34), while MCI also predicts later high-risk depression (SHR = 1.16; 95%CI: 1.01-1.33) in adults aged 50 years and older^6^.

The risk of both MCI and dementia is increasing in adults with subjective cognitive impairment (SCI)^2^. Furthermore, individuals with SCI who also present with Alzheimer’s disease (AD) pathology (amyloid positivity with elevated p-tau or t-tau) are significantly more likely to develop MCI or dementia than those with SCI without AD pathology^7^.

SCI refers to the perception of cognitive decline - typically involving memory - in the absence of objectively measurable deficits. Several related constructs have been used to describe SCI, such as subjective memory complaints, perceived forgetfulness, or cognitive concerns^8^.

SCI represents a heterogeneous condition that can evolve differently over time, depending on the underlying causes. To identify potential causes in a given individual, it is essential to assess cognitive concerns while considering key aspects such as the affected cognitive domains, the presence of specific worries, the onset of symptoms, possible associations with physical or mental health conditions, and potential links to medication, alcohol, or other substance use^2^.

Even in the absence, the subjective experience of cognitive decline among older adults is increasingly relevant in clinical practice, as more individuals seek advice for these concerns^8^. The prevalence in SCI is estimated at 1.5-15.5%, and over 60% of affected individuals exhibit neuropsychiatric symptoms^9^. SCI reflects affective symptoms, such as depression and anxiety, rather than true cognitive dysfunction^10^ and it increases the risk of progression to MCI^11^. Characterizing affective symptoms among people with SCI is therefore crucial to identifying individuals at risk and implementing appropriate non-pharmacological interventions^8^. The lower the degree of cognitive impairment, the greater the neuroplastic potential and learning capacity, and the higher the likelihood of inducing neurogenesis. Consequently, cognitive interventions should be implemented at the earliest stages of cognitive decline^12^.

Cognitive stimulation (CS) is a non-pharmacological intervention recommended in guidelines and widely implemented internationally^13^. It consists of non-specific cognition-enhancing activities, often conducted in a group setting^14^. CS differs from other approaches such as cognitive training and cognitive rehabilitation by its broader focus and social component, aiming to improve both mood^13^ and cognitive function^13^^,^^14^. Previous research has suggested that the efficacy of computerized CS on cognitive, emotional, or psychosocial outcomes warrants further investigation^15^.

Leisure activity is defined as the voluntary use of free time for pursuits outside of daily routines and is considered an essential component of a healthy lifestyle^16^. Both physical (RR 0.87; 95% CI 0.78-0.96) and cognitive (RR 0.66; 95% CI 0.52-0.85) leisure activities have been associated with a reduced risk of AD^17^. Stimulating leisure activities (SLA) may help individuals accumulate neural resources throughout life to buffer against cognitive decline^18^. Promoting social participation through a greater availability and diversity of community-based activities enables individuals to select those best suited to their interests^19^.

Building on this evidence, the present study aimed to evaluate the effects of a personalized, short-term, computerized CS program implemented in primary care on global cognition, memory, activities of daily living, and symptoms of depression and anxiety, compared with stimulating leisure activities, in community-dwelling adults aged 50 years and older with SCI or MCI.

## METHODS

This study was a randomized, controlled, single-blind trial.

The study population included adults aged ≥50 years with SCI or MCI. All participants were recruited from primary care consultations in Zaragoza (Spain). Exclusion criteria were: (1) use of acetylcholinesterase inhibitors (as these may affect global cognition and/or cognitive function); (2) significant sensory deficits, agitation, or aggressive behaviour (which would hinder participation in the interventions); and (3) prior participation in CS or memory workshops within the past 12 months.

The complete study protocol, including recruitment, enrolment, randomisation, and blinding procedures, has been published previously (Gómez- Soria et al. 2025)^20^.

This study was approved by the Ethical Committee of Clinical Studies of Aragón (Act No. 26/07/2023; study registration number PI23/368) and registered at ClinicalTrials.gov (Identifier: NCT06058611).

### Intervention procedures

*Intervention group 1 (IG1): Computerized cognitive stimulation*

Participants in IG1 were trained to use the computerized CS platform *Stimulus*. A short introductory session explained the cognitive neuroconstructs to be targeted and provided guidance from home-based training. IG1 participants performed personalized/adapted computerized CS at home through the *Stimulus* platform for 30 minutes per day, 5 days per week, over 8 weeks (total: 40 sessions). Further details are available in the published protocol^20^. The following neuroconstructs were trained: short-term and long-term memory, language, calculation, perception, logical reasoning, attention, executive functions, processing speed, and visual-motor skills. The use of external aids such as clocks and calendars was also encouraged.

The computerized CS program was adapted to participants´ cognitive level, as assessed by the MEC-35, and the difficulty was dynamically adjusted based on performance an available time. The intervention was further personalized to accommodate participants´ schedules.

Attendance at in-person sessions and notes on difficult exercises were recorded in a monitoring diary.

*Intervention group 2 (IG2): Stimulating leisure activities*

This intervention was led by trained professionals through three in-person sessions. IG2 participants engaged in two to five cognitively stimulating leisure activities per week for 8 weeks. Participants recorded the types of activities performed weekly and their daily frequency (<30 min, 30 min-1 h, 1-2 h, >2 h)^20^.

**Figure 1:**
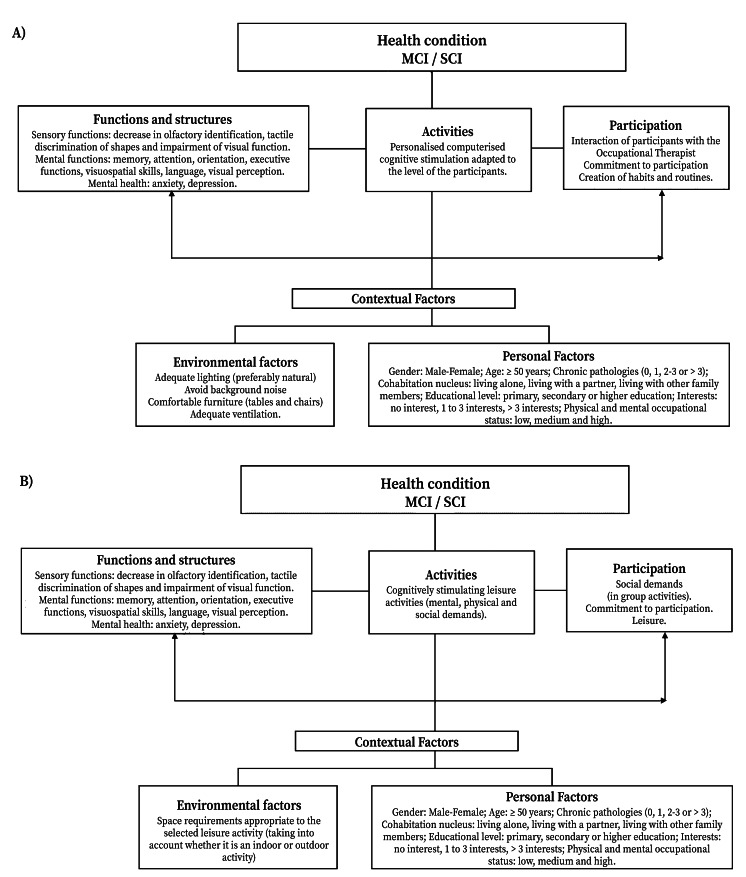
Health condition referenced according to ICF and OPTPF 4, focusing on personalized/adapted computerized cognitive stimulation (**A**) and cognitively stimulating leisure activities (**B**).

The intervention framework was based on the International Classification of Functioning, Disability, and Health (ICF) and Occupational Therapy Practice Framework 4th edition (OTPF-4) (Fig. 1).

*Control group*

The control group (CG) did not received either intervention (CS or SLA) during the study period. However, they participated in a 1.5-hour educational session covering risk and protective factors for cognitive decline, followed by practical memory enhancement strategies.

### Assessments

Data collection occurred at three time points: pre-intervention, post-intervention, and six-month follow-up. Variables included sociodemographic, clinical, and lifestyle factors. While general procedures are detailed in the study protocol^20^, this article expands information on the clinical and lifestyle variables considered:

- Clinical characteristics: number of chronic conditions (none, 1 - 3, >3); alcohol consumption (for men, ≥5 drinks on any day or ≥15 per week; for women, ≥4 drinks on any day or ≥8/week)^21^; treatment for depression; and treatment for anxiety.
- Lifestyle variables: smoking (≥10 cigarettes per day for at least two years)^22^; physical activity (sedentary-light, moderate, or vigorous) assessed using the International Physical Activity Questionnaire (IPAQ)^23^, and adherence to a Mediterranean diet.

*Primary and secondary variables*

The primary outcome was cognitive performance, assessed using the MEC-35, the Spanish version of the Mini-Mental State Examination (MMSE).

Secondary outcomes covered multiple cognitive, functional, and emotional domains, assessed using the following instruments: Memory Impairment Screen (T@M), the Set-Test (S-T), the Technology-Activities of Daily Living Questionnaire (T-ADLQ), the Lawton and Brody (L-B) scale, the 15-item Geriatric Depression Scale (GDS-15) (Yesavage), and the Goldberg Anxiety Subscale.

Additionally, participants completed the questionnaire “Cognitively stimulating cognitive leisure activities: scoring based on its three components at different stages of the life”, adapted from Karp et al., 2006^24^.

*Sample size*

Using a mixed between-within subjects repeated-measures ANOVA and data from Gómez Soria et al.^25^ for MEC-35 scores in the mildly impaired group, (*n2/p*=0.864), with α<0.05, power 85%, and a 15% anticipated dropout rate, a total sample of 59 participants was required.

### Statistical analysis

Qualitative variables were described as absolute frequencies and percentages, and quantitative variables as means and standard deviations. The Shapiro-Wilk test was applied within each group to assess variable distribution. Baseline differences were examined using the Kruskal-Wallis H test for quantitative variables and Fisher’s exact test for qualitative variables.

Outcome variables were analysed using linear mixed-effects models with Restricted Maximum Likelihood (REML) estimation. Subjects were modelled as random effects, and the time*group interaction was modelled as a fixed effect, controlling for age when significant differences were detected. Given the small sample size and the non-normal distribution of outcome variables, the Kenward-Roger correction was applied to adjust the degree of freedom, and p-values were obtained via permutation tests^26^. Effect sizes were estimated using the R^2^ of Nakagawa & Schielzeth^27^. For variables showing significant differences, *post hoc* tests with Bonferroni correction were applied while adjusting for age, and marginal means were subsequently computed. A complementary analysis was performed within the leisure activity group to explore potential differences in outcome variables according to the predominance of the mental component, using a similar linear mixed model.

All statistical analysis were conducted in R software, version 4.1.3 program (R Foundation for Statistical Computing, Institute for Statistics and Mathematics, Welthandelsplatz 1, 1020 Vienna, Austria). The significance level was set at p<0.05.

## RESULTS

**Figure 2:**
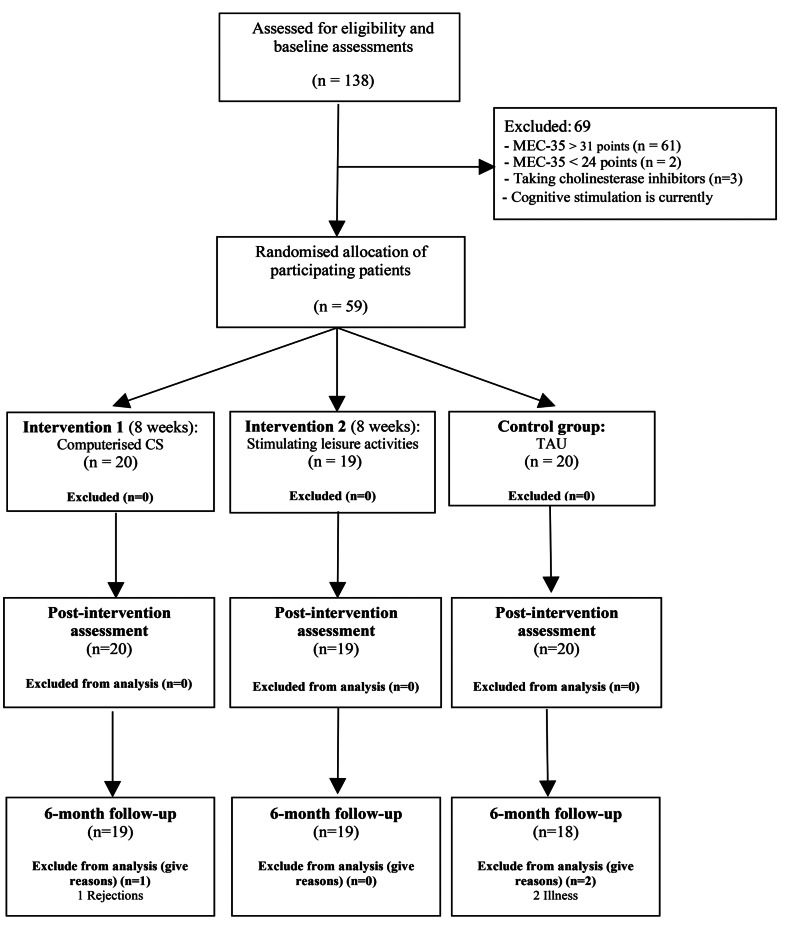
Consort 2001. Flow diagram.

CS: cognitive stimulation; TAU: usual treatment.

This study included 59 older adults; 20 participants were allocated to IG1, 10 to IG2, and 19 to the CG (Fig. 2).

The groups were comparable except for age, which was lower in IG1, and for the prevalence of heart disease, which was higher in IG1 than in the other groups, although this difference did not reach statistical significance (Table 1). According to scores on the MEC-35, 44 participants obtained between 28 and 31 points, which could indicate SCI (high cognitive level), and 15 obtained 24 to 27 points, which could indicate MCI (low cognitive level).

**Table 1:** Sociodemographic and clinical characteristics of participants by group

Variables	IG 1	CG	IG 2	p value
n (%)	(n= 20)	(n= 20)	(n=19)	*X*^*2*^
*Socio-demographic characteristics*				
Age, mean ± SD	66.65±10.82	73.45±9.70	75.58±10.03	0.028*
Gender (female)	15 (75.00)	14 (70.00)	12 (63.20)	0.728
Study level				0.194
Complete primary schools	4 (20.00)	4 (20.00)	3 (15.80)	
Higher education	8 (40.00)	3 (15.00)	5 (26.30)	
Incomplete primary schools	6 (30.00)	13 (65.00)	11 (57.90)	
University studies	2 (10.00)	0 (0.00)	0 (0.00)	
Physical Occupation				0.314
High	7 (35.00)	6 (30.00)	5 (26.30)	
Low	8 (40.00)	3 (15.00)	6 (31.60)	
Medium	5 (25.00)	11 (55.00)	8 (42.10)	
Mental Occupation				0.253
High	11 (55.00)	9 (45.00)	4 (21.10)	
Low	2 (10.00)	4 (20.00)	5 (26.30)	
Medium	7 (35.00)	7 (35.00)	10 (52.60)	
Family Nucleus				0.170
Live with friends	3 (15.00)	1 (5.00)	0 (0.00)	
Lives alone	4 (20.00)	5 (25.00)	6 (31.60)	
Lives with a partner	9 (45.00)	14 (70.00)	10 (52.60)	
Lives with other relatives	4 (20.00)	0 (0.00)	3 (15.80)	
Mediterranean diet	17 (85.00)	20 (100.0)	16 (84.20)	0.199
Interests				
1- 3	11 (55.00)	6 (30.00)	7 (36.80)	0.272
≥3	9 (45.00)	14 (70.00)	12 (63.20)	
Roles				
1	3 (15.00)	1 (5.00)	1 (5.30)	0.358
≥3	16 (80.00)	16 (80.00)	13 (68.40)	
2	1 (5.00)	3 (15.00)	5 (26.30)	
Values				0.322
Personal	18 (90.00)	20 (100.00)	19 (100.00)	
Social	2 (10.00)	0 (0.00)	0 (0.00)	
*Clinical characteristics*				
Low cognitive level	5 (25.00)	6 (30.00)	4 (21.10)	0.930
Chronic pathologies				
Diabetes Mellitus	1 (5.00)	5 (25.00)	4 (21.10)	0.219
Hypercholesterolemia	6 (30.00)	7 (35.00)	9 (47.40)	0.557
Obesity	7 (35.00)	4 (20.00)	3 (15.80)	0.404
Heart disease	1 (5.00)	5 (25.00)	6 (31.60)	0.084
Anemia	3 (15.00)	1 (5.00)	0 (0.00)	0.310
Degenerative joint disease	5 (25.00)	2 (10.00)	4 (21.10)	0.498
Hearing deficit	9 (45.00)	8 (40.00)	5 (26.30)	0.495
Cerebrovascular disease	1 (5.00)	5 (25.00)	2 (10.50)	0.196
Stroke	0 (0.00)	4 (20.00)	3 (15.80)	0.125
Smoking	5 (25.00)	7 (35.00)	2 (10.50)	0.237
Physical Activity				0.740
Moderate	13 (65.00)	11 (55.00)	13 (68.40)	
Sedentary-Light	4 (20.00)	7 (35.00)	5 (26.30)	
Vigorous	3 (15.00)	2 (10.00)	1 (5.30)	
Depression				
Diagnosis	9 (45.00)	6 (30.00)	4 (21.10)	0.288
Treatment	7 (35.00)	6 (30.00)	4 (21.10)	0.675
Anxiety				
Diagnosis	6 (30.00)	4 (20.00)	3 (15.80)	0.630
Treatment	5 (25.00)	5 (25.00)	2 (10.50)	0.475

IG 1: intervention group 1 (computerized cognitive stimulation); CG: Control group; IG 2: intervention group 2 (stimulating leisure activities); SD: standard deviation; *: compared by Kruskal-Wallis test.

Table 2 provides the data from the participants’ follow-up diaries. Both intervention groups showed good adherence, with slightly higher attendance in IG2. In IG1, the exercises perceived as most difficult were those related to processing speed, either alone or combined with attention and executive functions, and participants predominantly engaged in leisure activities with a mental component. In contrast, participants in IG2 engaged in a wider variety of stimulating leisure activities - especially those involving physical, mental, and social components - and devoted more weekly time to cognitively demanding tasks. This greater engagement in stimulating activities appears to be related to their concern about memory failures.

**Table 2:** Participant monitoring diary IG 1 and IG 2

	IG 1 (n= 20)	IG 2 (n=19)
*Low cognitive level, n(%)*		5 (25.00)	4 (21.10)
*Attendance at face-to-face sessions, mean ±SD*		3.10±0.64	3.37±0.83
*Commitment in carrying out the exercises computerized, mean ±SD*		0.92±0.11	
*Difficulty in exercises computerized, n(%)*	Calculation	1 (5.00)	
	Processing speed	10 (50.00)	
	Attention	3 (15.00)	
	Attention, calculation	1 (5.00)	
	Attention, short-term memory	1 (5.00)	
	Calculation	1 (5.00)	
	Executive functions	2 (10.00)	
	Visuo-motor ability	1 (5.00)	
*Component in leisure time activities, n(%)*	Mental	14 (70.00)	
	Physical	6 (30.00)	
	Social	4 (20.00)	
*Physical Component*			
Predominance in the current leisure time, n(%)	No		13 (68.40)
	Moderate		3 (15.80)
	Low		10 (52.60)
	Yes (High)		6 (31.60)
Leisure activities, mean ±SD			1.37±0.60
Time (hours/week) dedicated to leisure activities, mean ±SD			13.63 ±7.75
*Mental Component*			
Predominance in the current leisure time, *n(%)*	No		9 (47.40)
	Moderate		3 (15.80)
	Low		5 (26.30)
	Not at all		1 (5.30)
	Yes (High)		10 (52.60)
Leisure activities, *mean ±SD*			2.37±1.16
Time (hours/week) dedicated to Leisure activities, *mean ±SD*			27.95±30.47
Social Component			
Predominance in the current leisure time*, n(%)*	No		15 (78.90)
	Moderate		1 (5.26)
	Low		10 (52.60)
	Not at all		4 (21.04)
	Yes (High)		4 (21.10)
Leisure activities, *mean ±SD*			1.26±1.05
Time (hours/week) dedicated to Leisure activities, *mean ±SD*			8.47±7.40

IG 1: intervention group 1 (computerized cognitive stimulation); IG 2: intervention group 2 (stimulating leisure activities); SD: standard deviation.

Significant time x group interaction effects were observed for the MMSE-35 (F_(4, 109.429)_=3.011, p=0.024), Goldberg index (F_(4, 109.607)_=4.56, p=0.004), and GDS-15 (F_(4, 109.631)_=3.22, p=0.023), with small but significant effect sizes and no baseline differences (Supplementary material, table 1). *Post hoc* comparisons showed significant differences between IG1 (cognitive program) and the CG in the MMSE-35 at 6-month follow-up and in the Goldberg index post-treatment, and between the CG and IG2 (leisure program) in the GDS-15 post-treatment and at 6-month follow-up (Supplementary material, table 2).

**Figure 3:**
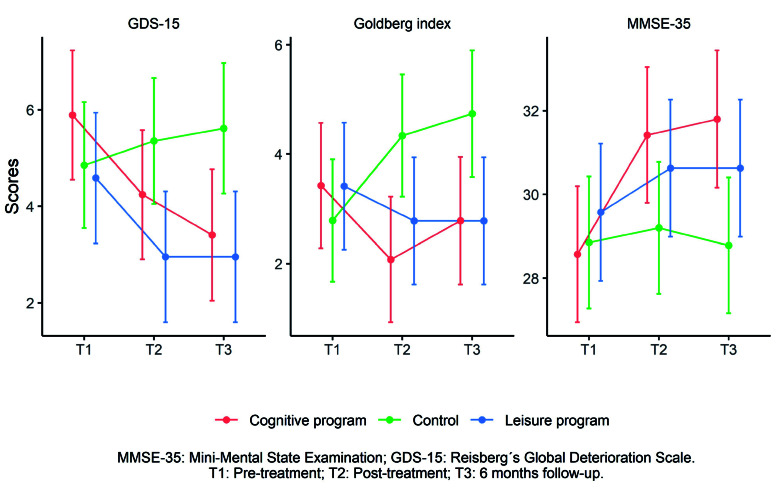
Between groups significant variables adjusted means at 6 months follow ups.

For variables showing significant differences, Bonferroni-corrected *post hoc* tests were applied, controlling for age**,** and adjusted marginal means for age were calculated. Age-adjusted GDS-15 scores were significantly lower in IG1 than in the CG at 6-month follow-up (3.407; 95%CI: 2.047-4.767 vs. 5.615; 95%CI: 4.262-6.968) and post-treatment for the Goldberg index (2.074; 95%CI: 0.927-3.222 vs. 4.338; 95%CI: 3.22-5.456). In contrast, for the MEC-35, the scores of the IG2 significantly outperform those of the CG, both post-treatment (30.626; 95%CI: 28.987-32.265 vs. 29.2; 95%CI: 27.625-30.776) and at 6-month follow-up (30.626; 95%CI: 28.987-32.265 vs. 28.782; 95%CI: 27.163-30.402) (Fig. 3).

There were no significant differences in the age of the participants in the leisure group according to the predominance of the mental component (p=0.162), so no model adjustment was required. No significant differences were observed in the leisure group between participants with and without predominance of the mental component across all measurements for the MMSE-35 (F_(2, 34)_=1.309, p=0.291), Goldberg index (F_(1, 17)_=1.035, p=0.335) , or GDS-15 (F_(2, 34)_=0.077, p=0.923).

## DISCUSSION

This randomized controlled trial demonstrates that personalized, short-term computerized cognitive stimulation intervention is associated with a significant reduction in anxiety levels post-intervention, as well as a decrease in depressive symptoms at the six-month follow-up. In contrast, the stimulating leisure activities program produced improvements in global cognition, both immediately after the intervention and at six-month follow-up.

However, we find no significant differences between the CS and stimulating leisure activities programs regarding memory and verbal fluency. Likewise, we observe no significant differences in ADL or global cognition in the computerized CS program, nor in mood improvements in the stimulating leisure activities program. Other studies have also reported no significant effects of computerized CS in verbal fluency^28^, no improvements in ADL through leisure activities^29^, and no significant memory improvements through stimulating leisure activities^30^ in individuals with MCI.

### Personalized computerized cognitive stimulation

There is significant post-intervention anxiety levels in younger and older adults with SCI and MCI. Jornkokgoud et al. (2024) similarly found that multidisciplinary team-delivered computerized CS reduced anxiety levels in older adults (60-75 years) with MCI after a short intervention (6 weeks, 30-minute session, once weekly) using multicomponent CS with or without multisensory integration in a hospital setting^31^. Other studies have also reported anxiety reduction through traditional CS in older adults (73.86 ±0.74 years) with SCI using adapted short, personalized intervention (45-minute sessions, once weekly for 10 weeks)^25^.

In contrast, other computerized CS studies in older adults with MCI reported no significant post-intervention anxiety improvements^28^^,^^32^ or improvements at three months^32^. These studies typically used extended programs (3 months, 12 sessions), longer sessions (between 90 minutes and one and a half hours), and lower weekly frequencies (once or twice weekly)^28^^,^^32^. 

We also observe a statistically significant reduction in depressive symptoms at six-month follow-up in young and older adults with SCI and MCI. Jornkokgoud et al. (2024)^31^ also reported decreased depressive symptoms after short-term multicomponent computerized CS with or without multisensory integration (6-week, 30-min/session, once a week) in older adults with MCI, although in their case improvements were detected post-intervention. A similar reduction was observed 12 months after traditional CS in older adults with SCI using a brief, adapted intervention (45-minute sessions, once a week for 10 weeks)^25^.

Therefore, our study provides a positive contribution to the evidence supporting computerized CS in this population. Maintenance CS therapy delivered by occupational therapy has been identified as the most cost-effective non-pharmacological intervention^33^ and the implementation of CS platforms is recommended for individuals with any level of cognitive impairment, except advanced dementia^14^. Such intervention may reduce healthcare costs and support more sustainability health systems^34^.

In addition, CS aligns closely with occupational therapy principles, including patient-centeredness, activity analysis, adaptation, and meaningful occupational engagemnet^35^.

### Stimulating leisure activities program

This program improves global cognition post-intervention and at six month follow-up. Doi et al. (2017)^30^ found similar results in older adults (mean age 76 years) with MCI after a longer (40 weeks) program based on dance (0.29; SD=2.6; p=0.026) and music activities (0.46; SD=2.1; p=0.008), with improvements in MMSE scores compared with a health education control group (-0.36; SD: 2.3) post-intervention. However, important occupational factors, such as personal interest, motivation, choice and control, habits, hobbies, personal sense of engagement, enjoyment and individual meaning -central to the conceptualisation of leisure activities^36^ - were not considered in their program. 

Participation in stimulating leisure activities have been shown to be beneficial for older adults with SCI, even without objective cognitive deficits^37^ and particularly in MCI^38^. Higher levels of leisure activity engagement are associated with reduced risk of developing MCI^39^. Furthermore, adapting activities to individual preferences can promote more positive attitudes toward leisure^40^.

Thus, our study provides relevant evidence for the role of stimulating leisure activities in improving cognition in older adults with SCI and MCI. Encouraging participation in such activities across the life span may be an important objective for public health an governmental prevention strategies^41^. Primary care is particularly well positioned both to identify older adults with memory concerns and to coordinate risk management after SCI or MCI has been identified^42^.

Further research is needed to directly compare computerized CS and stimulating leisure programs in randomised controlled trials with larger samples to determine which intervention is more effective for individuals with MCI and SCI. It would also be valuable to evaluate the combined approaches using computerized and traditional (with pencil and paper) CS delivered in brief sessions by multidisciplinary teams - including occupational therapists - in community health settings.

There are limitation to our study. One involves the use of new technologies, which, although beneficial for independence, quality of life, and well-being, may pose challenges for some older adults, particularly those with MCI^43^. Another limitation is the lack of information on MCI subtypes and the absence of standardised categories for stimulating leisure activities, which may affect reliability.

To minimise these limitations, we: 1) provided training before and during interventions to reduce technology-related difficulties, offering face-to-face sessions and phone support for questions at home; 2) personalised exercises according to cognitive level and affected domains despite not knowing MCI subtypes; and 3) recorded stimulating leisure activities weekly and categorized them into overall frequency levels (high, moderate, low) based on mental, physical, and social components.

In conclusion, this randomized controlled trial demonstrates benefits of personalized computerized CS for younger and older adults with SCI and MCI in community settings, including reduced anxiety and depressive symptoms. Participation in stimulating leisure activities improves global cognition. No significant differences are observed in memory, verbal fluency, or ADL. These findings have important clinical implications, offering potential avenues for reducing anxiety and depression through CS and slowing cognitive decline and potential progression to dementia through stimulating leisure activities.

## Data Availability

They are available upon request to the corresponding author.
